# Trends of cardiothoracic trauma at new trauma centre

**DOI:** 10.1186/1749-8090-8-S1-O74

**Published:** 2013-09-11

**Authors:** P Mhandu, S Chaubby, D Robb, M Uzzaman, H Khan, D Whitaker

**Affiliations:** 1 Kings College Hospital, London, UK

## Background

To analyse the demographics, types and mechanisms of injury, management and outcomes of all cardiothoracic trauma during the first year of a South London Major Trauma Centre in comparison to national standards.

## Methods

A retrospective analysis of a trauma database in conjunction with electronic patient records and paper notes for the 12 month period April 2010 to March 2011.

## Results

Of 1556 trauma patients, 254 had cardiothoracic trauma. 90% (228/254) were male and 10% (26/254) were female. Median age of all patients was 26 (range 1-91). 57% (145) were penetrating injuries mainly knife wounds (128) and gunshots (10). 43% (109) were blunt injuries (90 high and 19 low velocity). The actual injuries are detailed with rib fractures (35%) and pneumothorax (30%) the commonest and cardiac (5%) and diaphragm (3%) injuries the least common. 48% (121) of all cases had isolated thoracic injury, with 52% (133) being multiply injured. Of those multiply injured 36% (48) had head injuries, 65% (87) had orthopaedic injuries and 32% (43) had abdominal injuries. Operative and non-operative management is described in detail. Of the 15 patients requiring cardiothoracic surgery, 6 had a clamshell incision, 5 posterolateral thoracotomy, 2 median sternotomy and 1 thoracoscopy. Overall mortality was 3.5% (9/254). Operative mortality was 13% (2/15). Median length of stay was 4 days.

## Conclusion

Despite a higher incidence of violent penetrating trauma compared to the national average of 2% of all thoracic trauma, the pre hospital care and in hospital multi-disclipinary approach with resident cardiothoracic care results in a favourably low mortality.

